# Broad-scale changes in lesser prairie-chicken habitat

**DOI:** 10.1371/journal.pone.0304452

**Published:** 2024-05-31

**Authors:** Megan P. Vhay, David A. Haukos, Daniel S. Sullins, Mindy B. Rice

**Affiliations:** 1 Kansas Cooperative Fish and Wildlife Research Unit, Kansas State University, Manhattan, Kansas, United States of America; 2 U.S. Geological Survey, Kansas Cooperative Fish and Wildlife Research Unit, Manhattan, Kansas, United States of America; 3 Department of Horticulture and Natural Resources, Kansas State University, Manhattan, Kansas, United States of America; 4 U.S. Fish and Wildlife Service, Fort Collins, Colorado, United States of America; Auburn University, UNITED STATES

## Abstract

Lesser prairie-chicken (*Tympanuchus pallidicinctus*) populations of in the Sand Sagebrush Prairie Ecoregion of southwest Kansas and southeast Colorado, USA, have declined sharply since the mid-1980s. Decreased quality and availability of habitat are believed to be the main drivers of declines. Our objective was to reconstruct broad-scale change in the ecoregion since 1985 as a potential factor in population declines. We assessed temporal change from 1985–2015 in landcover types and calculated landscape metrics using Land Change Monitoring, Assessment, and Projection imagery layers. We also documented presence of anthropogenic structures including oil wells and electrical transmission lines. Landcover type composition changed little since 1990 across the Sand Sagebrush Prairie Ecoregion. However, anthropogenic structures (i.e., oil/gas wells, cell towers, wind farms, and transmission lines) notably increased, potentially causing functional habitat loss at a broad scale. Increased anthropogenic structures may have decreased habitat availability as well as the quality of existing habitat for lesser prairie-chickens, possibly contributing to recent population declines throughout the Sand Sagebrush Prairie Ecoregion.

## Introduction

Habitat loss and fragmentation are major drivers of declining biodiversity in ecosystems worldwide [1]. Among the most threatened ecosystems are grasslands, which are often reduced by conversion of land for row-crop agriculture [2] as well as infrastructure development to meet ever-increasing energy demands [3–5]. Such landscape changes in North America have contributed to an estimated loss of 70% of Great Plains grasslands [6] and reduced habitat available for the region’s wildlife [7].

While some generalist Great Plains species including coyote (*Canis latrans*) and white-tailed deer (*Odocoileus virginianus*) continue to thrive despite widespread landscape change [8], other species including many grassland birds have declined during the past 50 years [9]. Among them, prairie grouse once numbering in the millions have been reduced to a fraction of their historic occupied range and population abundance [10,11]. Prairie grouse in the Great Plains (*Tympanuchus* spp.) are inherently dependent on large grassland-dominated landscapes to complete their life histories and are considered indicators of grassland ecosystem integrity [10,11]. Reliance on extensive heterogeneous grasslands indicates the potential for prairie grouse to serve as focal species for other grassland wildlife [10,12].

**Fig 1:**
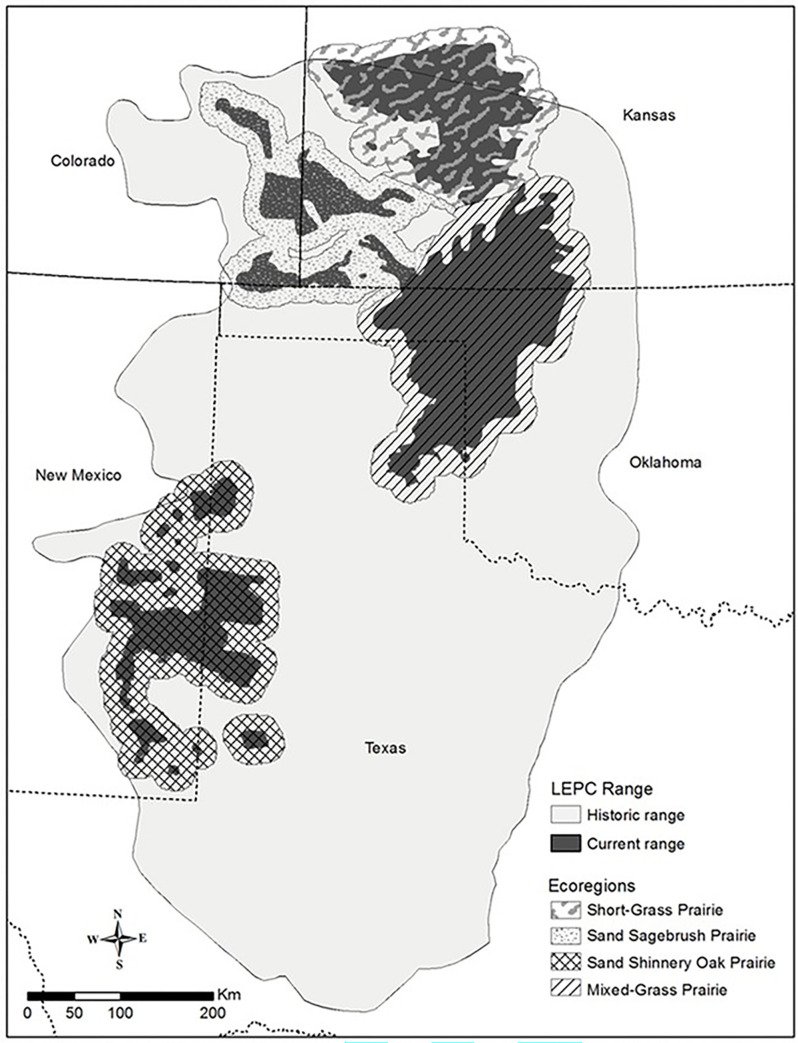
Lesser prairie-chicken range. Presumed historical and current distribution, as well as current ecoregion boundaries, for lesser prairie-chickens (LEPC) in the Southern Great Plains, USA. Figure adapted from Boal and Haukos [11].

The lesser prairie-chicken (*T*. *pallidicinctus*) is a prairie grouse that inhabits portions of the southwestern Great Plains, a region represented by sandy soils and mid- to tall grasses or native shrubs (e.g., sand sagebrush [*Artemisia filifolia*] and sand shinnery oak [*Quercus havardii*]) within the larger expanse of short-grass or mixed-grass prairie [13,14]. Currently, lesser prairie-chickens occur in four distinct ecoregions (Short-Grass Prairie/CRP Mosaic, Mixed-Grass Prairie, Sand Sagebrush Prairie, and Sand Shinnery Oak Prairie) across five states (Kansas, Colorado, Oklahoma, Texas, and New Mexico [15]; Fig 1). The lesser prairie-chicken has persisted through numerous environmental and anthropogenic events, including intensive drought (e.g., 1930s and 1950s) and loss of intact prairie to center-pivot irrigation agriculture since the 1960s [11,14,16,17]. Despite their historical resilience, populations have declined range-wide since the mid-1980s [11]. In November 2022, the U.S. Fish and Wildlife Service declared the lesser prairie-chicken in the southern Sand Shinnery Oak Prairie Ecoregion population segment as endangered, and the northern population segment containing all other ecoregions as threatened [18].

Within the northern population segment, the Sand Sagebrush Prairie Ecoregion of southwestern Kansas and southeastern Colorado hosted the greatest population of lesser prairie-chickens for much of the past 50 years. Lesser prairie-chicken populations of the Sand Sagebrush Prairie Ecoregion historically fluctuated in response to extreme environmental conditions (e.g., prolonged drought; winter storms) but remained resilient overall [19]. An estimated >86,000 birds occupied the region during the 1970s and early 1980s [20,21]. However, populations decreased dramatically after the mid-1980s, and currently the Sand Sagebrush Prairie Ecoregion has the second-lowest density and abundance of lesser prairie-chickens among the five ecoregions. Estimates from aerial surveys were only 440 and 1,713 lesser prairie-chickens in 2021 (90% CI = 56, 1,012) and 2022 (90% CI = 209, 3,861), respectively [22].

Throughout changes to legal and regulatory status, conditions limiting lesser prairie-chickens largely remain. Estimates of prairie area required for lesser prairie-chicken population persistence range from 4,900 ha to >20,000 ha [13]; the U.S. Fish and Wildlife Service recommend a minimum of 10,118 ha of high-quality habitat [23]. It is also estimated that lesser prairie-chickens require landscapes comprised of at least 63% grassland to support large, stable populations [24], though peak probability of use has been observed at approximately 77% grassland [25] with resilience to intensive drought maximized at 90% grassland [26]. Below these thresholds, populations will be greatly diminished or become locally extinct. Land cover change coupled with increased drought frequency and intensity may have negatively affected continued resilience to drought events in the semi-arid portion of the lesser prairie-chicken range, including the Sand Sagebrush Prairie Ecoregion [19,26,27].

The dominant factor(s) driving the contemporary decline of lesser prairie-chickens in the Sand Sagebrush Prairie Ecoregion has not been identified; however, changes in habitat are thought to be responsible [10,19,28]. Although landcover did not change on the U.S. Forest Service Cimarron and Comanche National Grasslands, conversion of native sand sagebrush prairie for row-crop agriculture was historically widespread throughout private lands in the region before the 1960s [11,14]. Scattered re-establishment of grasslands began after 1985 through the U.S. Department of Agriculture Conservation Reserve Program (CRP), which incentivized converting highly erodible crop fields to perennial grassland under contract periods lasting ≥10 years [29]. While establishment of CRP essentially offset grassland losses from the 1950s through 2014 in the lesser prairie-chicken range across Kansas [14], lesser prairie-chickens in the Sand Sagebrush Prairie continued to decline during this period, suggesting that total grassland area may not have driven population trends.

Other broad-scale changes independent of vegetation cover may have contributed to lesser prairie-chicken population declines in the Sand Sagebrush Prairie Ecoregion. Lesser prairie-chickens inherently avoid large vertical features on the landscape, resulting in spaces of unavailable habitat varying in distance by structure type [3,25,30,31]. It is hypothesized that both trees and vertical anthropogenic structures are perceived by lesser prairie-chickens as perches for aerial predators [32,33]. Although tree occurrence was not found to have changed in our study area [34], functional habitat loss and fragmentation could be expected due to avoidance response by lesser prairie-chickens to widespread anthropogenic infrastructure. Such changes in habitat availability, in addition to possible change in prairie-dominated areas, may have driven declines of lesser prairie-chickens at a broad scale.

Previous research addressed broad-scale landcover change in the Kansas extent of the lesser prairie-chicken occupied range since 1950 [14,35]. However, a longitudinal retrospective analysis of other broad-scale changes for the entire Sand Sagebrush Prairie Ecoregion was needed to complement those efforts. We hypothesized that anthropogenic structures increased, total native prairie and area of prairie patches decreased, and cropland increased, all of which would negatively affect population abundance and distribution of lesser prairie-chickens in the ecoregion by decreasing available habitat needed to support the species. We assessed the change in number of vertical anthropogenic structures (i.e., oil and gas wells, wind turbines, transmission lines, and cell towers) present in across the ecoregion. We also measured change in landscape composition and configuration of private lands in the ecoregion from 1985–2015.

## Materials and methods

### Study area

**Fig 2:**
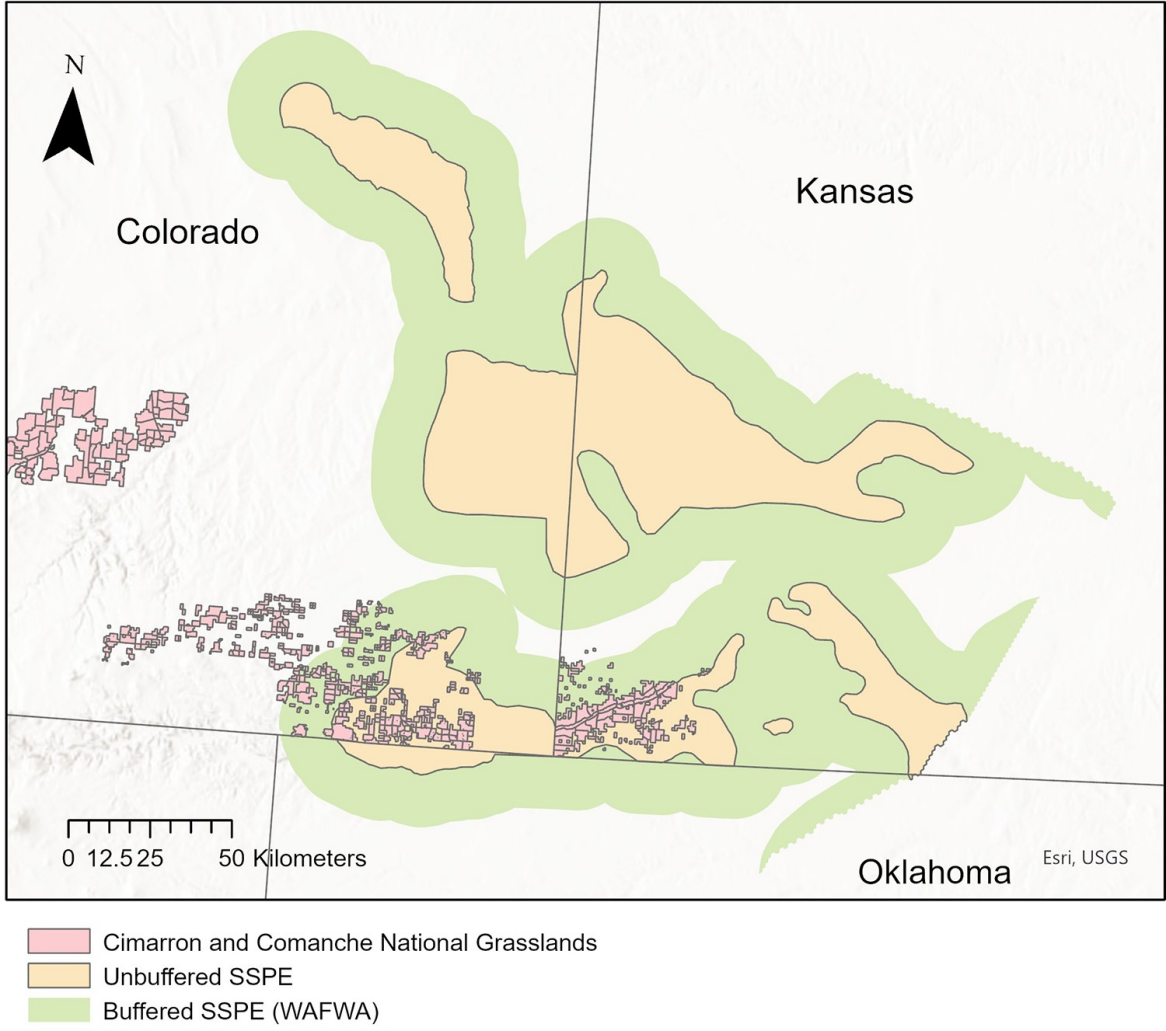
Study area. The Sand Sagebrush Prairie Ecoregion (SSPE) within the estimated occupied range of the lesser prairie-chicken, including U.S. Forest Service Cimarron (Kansas) and Comanche (Colorado) National Grasslands, USA. Shown are both the SSPE area as delineated by McDonald et al. [15] and the boundary updated with a 16 km by the Western Association of Fish and Wildlife Agencies (WAFWA; [36]). Copyright ©2016 from Ecology and Conservation of Lesser Prairie-Chickens edited by David A. Haukos and Clint W. Boal. Reproduced by permission of Taylor and Francis Group, LLC, a division of Informa pic. Figure used for illustrative purposes only.

We classified landcover types within the Sand Sagebrush Prairie Ecoregion, one of four ecoregions delineated by McDonald et al. [15] and Van Pelt et al. [36], comprising the estimated occupied range of the lesser prairie-chicken in the southern Great Plains ([11]; Fig 1). We defined our study area using the boundary modified by Western Association of Fish and Wildlife Agencies (WAFWA), which added a 16-km buffer around the 2013 estimated occupied range ([36]; Fig 2). The study area spanned approximately 32,516 km^2^ in 21 counties of southwest Kansas, southeast Colorado, and northern Oklahoma panhandle, including both public sand sagebrush prairie (U.S. Forest Service Cimarron and Comanche National Grasslands) and private lands. The latter were composed primarily of grazed working grasslands, U.S. Department of Agriculture CRP grasslands, and row-crop agriculture [37]. Grazing intensity in the region was typically moderate to high, with grazing pressure on the Cimarron National Grasslands having nearly doubled since the mid-1950s [28].

Bands of sandy soils such as those running parallel to the Cimarron and Arkansas River drainages, characteristic of the sand sagebrush prairie, supported the eponymous sand sagebrush shrubs. Also present were mid- to tall grasses including big bluestem (*Andropogon gerardii*), little bluestem (*Schizachyrium scoparium*), sideoats grama (*Bouteloua curtipendula*), and sand dropseed (*Sporobolus cryptandrus*; [19]). Short-grass prairie species, supported in clayey and loamy soils, included blue grama (*Bouteloua gracilis*) and buffalograss (*B*. *dactyloides*; [28]). Common forbs in the ecoregion included annual buckwheat (*Eriogonum annuum*), blazing star (*Liatris* spp.), western ragweed (*Ambrosia psilostachya*), prairie sunflower (*Helianthus periolaris*), annual sunflower (*H*. *annuus*), Russian thistle (*Salsola tragus*), camphorweed (*Heterotheca subaxillaris*), Indian blanket flower (*Gaillardia pulchella*), tansy aster (*Machaeranthera tanacetifolia*), buffalo bur (*Solanum rostratum*), buffalo gourd (*Cucurbita foetidissima*), wax goldenweed (*Grindelia papposa*), prickly lettuce (*Lactuca serriola*), and marestail (*Conzya canadensis*; [28]).

Initially, CRP fields in Kansas and Colorado were seeded with a native grass mixture; seed mixtures did not include forbs until the mid-1990s [17]. Historically, CRP grasses in Kansas were limited to native warm-season species such as little bluestem, sideoats grama, and switchgrass (*Panicum virgatum*). Other grass species in CRP throughout the ecoregion included big bluestem, western wheatgrass (*Pascopyrum* spp.), blue grama, buffalograss, and Indiangrass (*Sorghastrum nutans*). Forbs in CRP fields included alfalfa (*Medicago sativa*), sweet clover (*Melilotus* spp.), Maximillian sunflower (*Helianthus maximiliani*), prairie bundleflower (*Desmanthus illinonensis*), purple prairie-clover (*Dalea purpurea*), and upright prairie coneflower (*Ratibida columnifera*; [28]).

Average annual precipitation for Elkhart, Kansas, in Morton County from 1900–2021 was 46 cm, varying from 23 to 76 cm (data from High Plains Regional Climate Center 2023). Drought in this region occurred on a roughly 5-year cycle [19].

### Broad-scale data

We quantified change over time for several broad-scale factors in the Sand Sagebrush Prairie Ecoregion to assess potential effects on lesser-prairie chicken populations. We quantified the total number of wind turbines, cellular/radio towers, transmission lines, and oil and gas wells in 5-year intervals from 1985 (the approximate point of contemporary lesser-prairie-chicken population peak) through 2015 to determine change in frequency of those structures over time.

To measure changes in the dominant vegetation landcover types on private lands, we quantified the area of cropland, native prairie (cover class “grass/shrub” with CRP omitted), and CRP in 5-year time steps. Finally, we estimated change in the area and number of large prairie patches (5,000 ha to >20,000 ha), as well as proportion of prairie at the 5-km scale, to identify changes in availability of potential habitat to support lesser prairie-chickens. We classified time steps for all anthropogenic features in intervals beginning with “pre-1985”, continuing in 5-year intervals from “1985–1989” (i.e., January 1, 1985 through December 31, 1989) through “2010–2014” to assign features to one of the time intervals. We referred to the recorded spud, completion, on-line, or construction dates (dependent on feature type) to determine when features appeared on the landscape. We then summed up the total number of each feature by time step to determine the change in structures over time. We worked in WGS84 projection when mapping spatial data.

#### Wind turbines

Wind turbine locations were available from the US Geological Survey’s US Wind Turbine Database for 1985–2019 [38].

#### Cellular towers

We obtained Kansas cellular/radio tower locations from Kansas Data Access and Support Center [39], a dataset based on Federal Communications Commission (FCC) Antenna Structure Registration. For Oklahoma and Colorado, we downloaded tower locations from the Homeland Infrastructure Foundation Level Data (HIFLD) open data site from the Department of Homeland Security as provided through ESRI arcgis.com [40]. We used “Location Buildout Deadline”, “Location Buildout Notification Date” or “Five Year Buildout Date”, in that order of availability as found in the cell tower FCC registry [41] to identify when towers were built.

#### Transmission lines

We obtained data for electrical transmission lines (defined on the HIFLD site as ranging from 69 kV-765 kV) for Oklahoma, Kansas, and Colorado from the HIFLD open data site as provided through ESRI arcgis.com [42]. We referred to Google Earth Pro and its historical imagery tool to estimate the time step in which each transmission line segment appeared on the landscape. Lines that we estimated as having been “constructed” during the late 1980s through mid-1990s in Oklahoma and Colorado likely predate the late 1980s, but fine-scale features are indistinguishable in Google Earth imagery prior to 1991. For Kansas lines, we cross-referenced our estimated dates with a shapefile from Sunflower Energy, allowing us to categorize many of the older Kansas lines as “pre-1985”. Where structures appeared to have been updated but the path of the line itself remained the same, we listed the time period of the original structure. We calculated the change in total length (km) of transmission lines at each time step.

#### Oil and gas wells

We obtained Colorado oil and gas well data from the Colorado Oil and Gas Conservation Commission (COGCC) WELLS shapefile in the COGCC COGIS Database [43]. We referenced the COGCC WELLS shapefile metadata and the Orphan Well Program page for attribute definitions [44]. From the clipped Sand Sagebrush Prairie Ecoregion set of 2,365 wells, we deleted 165 entries classified as “Abandoned Location” (AL; meaning the operator permitted the project, but no construction took place [45]), as well as 130 wells marked as “Dry and Abandoned” before 1985 (DA facility status, with a status date before 1985). We also omitted 42 records lacking spud dates and start dates; nearly all were classified by Location Qualifier “Planned Footage” or “Planned Lat/Long”, with the label “Planned” indicating that a well was never drilled. After removing these entries from the set, there remained 2,006 wells in the Colorado portion of the ecoregion as of December 2014.

We sourced Kansas well locations from a Kansas Geological Survey data set available through the Kansas Data Access and Support Center [46]. From the original set of 27,010 records for the SSPE, we omitted 931 entries with the Well Class “Expired Intent to Drill”, and entries lacking both a Spud and Completion date (2,123 records), as at least one date was needed for an approximate start date. We excluded wells plugged prior to 1985 to remain consistent with our deletions in the Colorado record. We did not include wells that appeared after 2015 to remain consistent with the temporal range of our landcover analysis. After removing these wells from the set, there were 20,864 wells in the Kansas component of the Sand Sagebrush Prairie Ecoregion as of December 2014.

Oklahoma well records came from the Oklahoma Corporation Commission web site [47]. We were unable to filter Oklahoma wells plugged before 1985 as done for Colorado and Kansas. Plug dates were not available in the Oklahoma data set; there were inconsistencies in classifiers (e.g., some AC [active] wells also being labeled “dry”), so we were conservative when deciding whether to remove wells. We removed duplicate points, leaving 302 well records for the Oklahoma portion of the Sand Sagebrush Prairie Ecoregion.

We removed “dry and abandoned” wells, those “plugged” prior to 1985 wherever possible, planned/pending wells lacking an apparent construction date, and entries labeled “abandoned location”. It should be noted that the wells we report are not necessarily active. Given lesser prairie-chicken avoidance of structures [31,48–50], our aim was to quantify number of structures on the ground, regardless of producing status. Partially due to identifier inconsistencies among data sets, and in many cases because neither plug dates nor any date indicating the end of an “active/producing” period was listed, we considered all wells as having a more or less permanent structure once established. It is possible that the total number of wells was slightly overestimated with this approach, as plugged wells (including around 4,700 wells in Kansas) may no longer have a pump jack present at the site. We estimated 23,167 extant wells present in the Sand Sagebrush Prairie Ecoregion as of 2014.

#### Landcover on private lands

We focused only on private lands in estimating landcover, as landcover represented by coarse vegetation cover class (grassland vs. cropland) was not expected to change over time on public lands (S8 File). We selected 3 dominant landcover types in the ecoregion: combined non-CRP grassland and shrubland (hereafter, “prairie” or “native prairie”); cropland; and CRP. We used data for these cover types to identify changes in total area and patch configuration for each cover type. We first identified “cropland” and “grass/shrub” cover types from Primary Land Cover rasters in 5-year intervals from 1985–2015 from LCMAP Collection 1.0 [51]. This collection was derived from Landsat surface data spanning 1985–2017, and characterized by a 30-m resolution.

We considered CRP as a separate cover type, given its important role in lesser prairie-chicken conservation as well as its impermanent status on the landscape [52]. The first plantings of CRP fields occurred in 1986, and took at least 4 years to mature [17]; we assumed that by 1990, CRP plantings had become established enough to constitute a separate cover class. However, accurate maps of CRP fields for the Sand Sagebrush Prairie Ecoregion were unavailable prior to 2014, and shapefiles of CRP enrollment were inconsistent among years, states, and counties. Additionally, the “grass/shrub” category in LCMAP did not differentiate between CRP and non-CRP grassland (i.e., prairie). We therefore estimated CRP cover across the ecoregion from 1990 through 2015 based on changes in LCMAP cropland and the “grass/shrub” cover type between years, and separated the “grass/shrub” into two cover classes: “native prairie” and “CRP” (S9 File). Using Raster Calculator in ArcGIS Pro (version 2.7), we identified changes in raster cell values from cropland to grassland, and vice versa, between consecutive time steps and used the difference in cell values to build a putative CRP cover class. During this process, we also added or removed CRP cells in succeeding steps (e.g., where cropland became grass/shrub, as well as when CRP fields expired and were converted to cropland). We then replaced all “non-CRP” cells in the newly created CRP rasters with the rest of the corresponding LCMAP landcover raster values for that year; this resulted in a new series of LCMAP rasters featuring CRP as an additional cover class.

We attempted to minimize the occurrence of isolated pixels inherent to the LCMAP raster classification, as these stray pixels in otherwise homogeneous fields would register as small “patches” and potentially inflate the estimated number of patches on the landscape. We applied the Majority Filter tool in ArcGIS Pro first to the original raster and then to the output raster of that run, repeating the process until pixels no longer appeared to change in a significant way; in total, 10 runs of the tool. Doing so incorporated isolated pixels of one cover type to match the surrounding cover based on the majority type represented in the 0.72-ha area of the 8 surrounding cells.

#### Landscape metrics

We used program FRAGSTATS to generate landcover metrics from private lands LCMAP-derived rasters for both composition and configuration of each dominant landcover type for each time step [53]. We used the default 8-cell neighborhood rule to determine patch membership for configuration metrics [35].

We calculated several landscape composition and configuration metrics for the private lands LCMAP rasters [14]. We used Contagion (CONTAG) to measure connectivity incorporating both the proportional abundance of each patch type in the study area landscape as well as the number of cell adjacencies for that patch type or another patch type as an index to broad-scale fragmentation [53]. We included Percentage of Landscape (PLAND), a composition metric, which reports the proportion of the study area composed of each cover type of interest, and allowed us to address change over time in landcover totals for the three dominant cover types. We used Mean Patch Area (AREA_MN) to find mean patch size in our landscape, and Number of Patches for the total number of patches for each cover type; we used these metrics to address changes in average area and connectivity, respectively, of grassland regions over time, which would be relevant to the lesser prairie-chicken’s requirement for grassland-dominated landscapes [11]. Our aggregation index was Clumpiness (CLUMPY), which incorporates like adjacencies and reflects the degree of clustering among patches for a given cover type [53]. We calculated Total Edge (TE) to measure the total length of patch edges for a cover type. Creating a separate private lands shapefile from the entire Sand Sagebrush Prairie Ecoregion may increase apparent total edge; however, using this raster boundary consistently throughout our analyses does allow for an accurate measure of change in edge throughout the time series. We chose both Clumpiness and Total Edge as a means of measuring isolation of patches over time.

All landcover metrics for CRP represent change since 1990 rather than 1985 to minimize the effect of CRP landcover category introduction on landcover metrics. Additionally, we provided only change since 1990 for configuration metrics, which incorporate the spatial arrangement of all cover types [53], as these would be affected by the introduction of the CRP cover type. PLAND was based on cover totals, independent of spatial arrangement [53]; therefore, we included 1985 (without the added CRP cover type) when calculating that metric.

#### Estimating change in large prairie areas

We assessed change in number of prairie patches based on area using FRAGSTATS patch-level output for native prairie cover. From the patch-level FRAGSTATS output, we identified the largest patches of prairie and categorized them into 3 categories: 5,000–9,999 ha, 10,000–19,999 ha, and ≥20,000 ha [13].

#### Percent prairie within 5-km scale

We conducted a moving window analysis to determine the proportion of prairie within 5 km at any given point on private lands (S10 File). We created a set of binary rasters (1 = grass/shrub; 0 = non-grass/shrub) from the LCMAP cover class rasters and used Focal Statistics in ArcGIS Pro to generate a raster image of 5-km windows. We excluded public lands, as well as windows bordering public lands from the analysis. From these outputs, we then created a time series of maps identifying areas as <30%, >30%, and >60% prairie within 5 km. We calculated the proportion of the landscape within each of the percent prairie categories.

## Results

**Fig 3:**
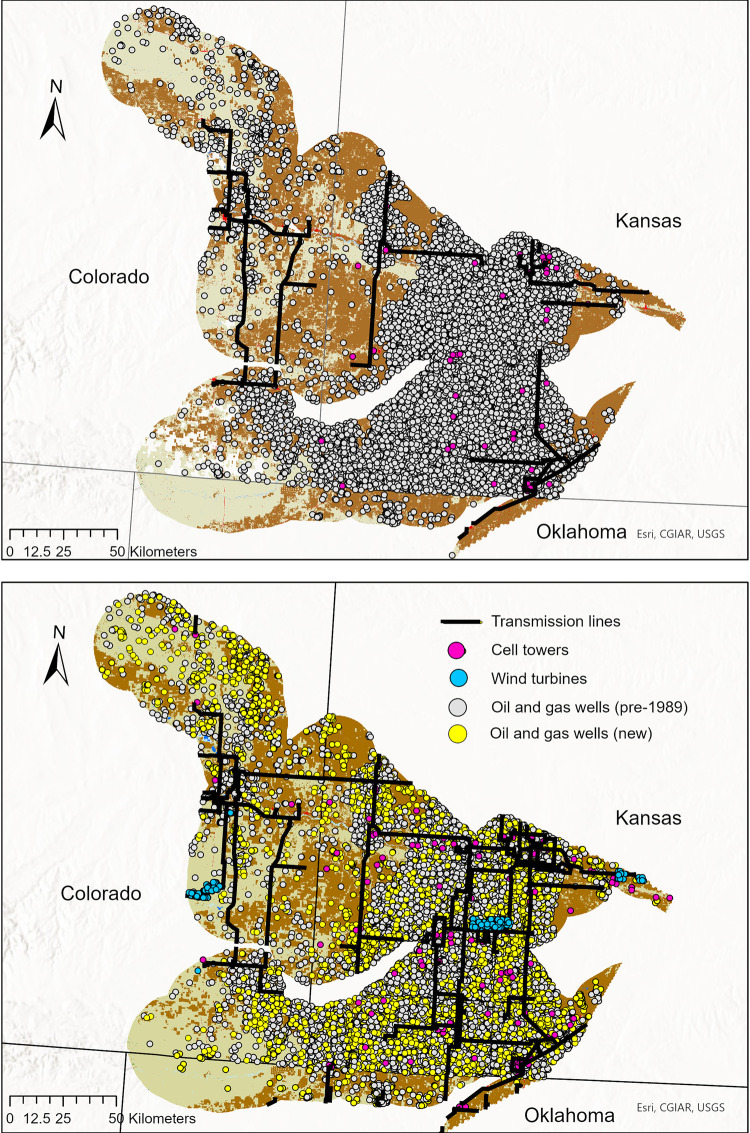
Anthropogenic structures on landscape. Number of anthropogenic structures (transmission lines, cell/radio towers, wind turbines, and oil/gas wells) present in the Sand Sagebrush Prairie Ecoregion of Kansas, Colorado, and Oklahoma, USA, in 1989 (top, approximate contemporary lesser prairie-chicken population peak) through the end of 2014 (bottom).

We found that all anthropogenic structure types increased since 1989 (Table 1 and Fig 3). The greatest increases in structures occurred mainly during the 1990s through mid-2000s.

**Table 1:** Anthropogenic structures. Total number or length of vertical anthropogenic structures present in the lesser prairie-chicken Sand Sagebrush Prairie Ecoregion (640,763 ha focal area plus 16-km buffer [36]) of southwest Kansas, southeast Colorado, and northwestern Oklahoma, USA, at 5-year intervals from 1989–2014.

Year	Oil/Gas Wells	Cell Towers	Wind Turbines	Transmission Lines (km)
1989	12,327	45	0	1134.27
1994	15,427	58	0	1552.05
1999	18,301	76	0	1819.00
2004	20,288	109	112	2316.04
2009	21,930	145	112	2443.46
2014	23,167	176	278	2582.06

Wind turbines increased by 166 structures (148.2%) from 2003 to 2014 (Table 1 and Fig 3). By the end of 2014, approximately 38.9% of turbines built since 1989 were located on grassland (either CRP or native prairie). Cell towers increased by 131 (291.11%) from 1989 to 2014 (Table 1 and Fig 3), with an estimated 34.7% of towers built since 1989 located in grassland. The total length of transmission lines increased by 1,447.79 km (127.6%) from 1989 to 2014 (Table 1 and Fig 3), with at least 28.10% of transmission lines crossing or immediately adjacent to grassland. Oil and gas increased by 10,840 wells (87.9%) from 1989 to 2014 (Table 1 and Fig 3). As of 2015, 31.1% of wells built since 1989 were located in grassland.

**Fig 4:**
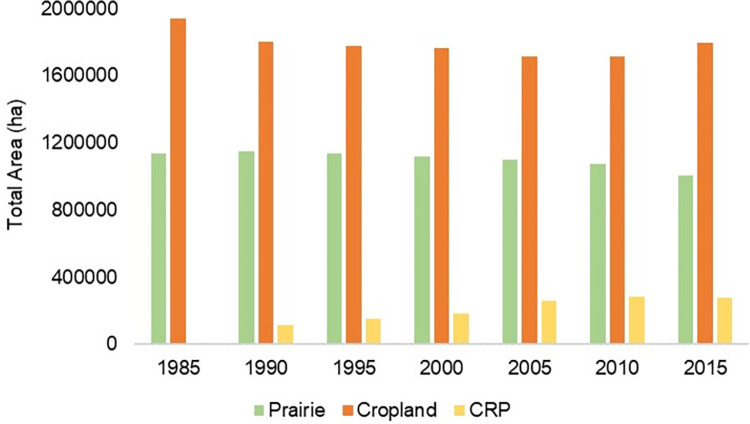
Total area of dominant cover types. Total area of each cover type (prairie, cropland, and Conservation Reserve Program [CRP] grassland) in the Sand Sagebrush Prairie Ecoregion of southwestern Kansas, southeastern Colorado, and northwestern Oklahoma, USA, in 5-year intervals from 1985–2015. The Conservation Reserve Program was initiated in 1986, and therefore excluded as an established cover type before 1990.

Landcover totals changed minimally from 1990–2015, with the exception of CRP introduction by 1990 (Fig 4). Total prairie on private lands decreased by 11.7% and cropland decreased by 7.3% since 1985 (Fig 4). Although total cropland decreased since 1990, this was concurrent with CRP increasing after the mid-1980s as enrollment across the Sand Sagebrush Prairie Ecoregion became more widespread (Fig 4). When combining prairie and CRP, total grassland increased by 1.1% from 1990–2015.

**Fig 5:**
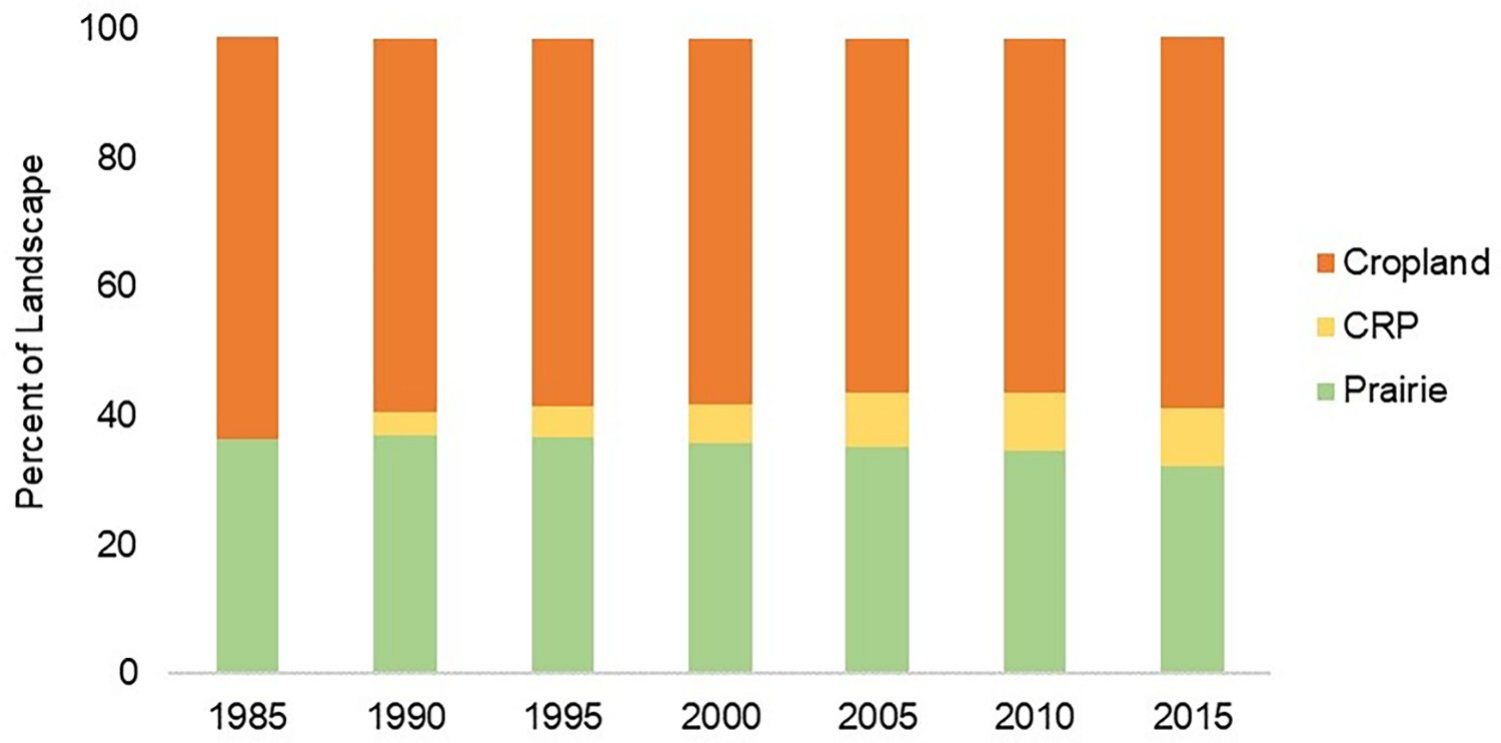
Proportions of dominant cover types. Proportions of the three dominant cover types (cropland, Conservation Reserve Program [CRP] grassland, and prairie) in the Sand Sagebrush Prairie Ecoregion of southwestern Kansas, southeastern Colorado, and northwestern Oklahoma, USA, from 1985–2015. The Conservation Reserve Program was initiated in 1986, and excluded from analysis as an established cover type before 1990.

Landscape composition of prairie changed over time. Percentage of Landscape consisting of prairie decreased by 4.3 percentage points from 1985 through 2015, while percent consisting of cropland decreased by 4.6 percentage points (Fig 5). From 1990 to 2015, the percent of landscape consisting of CRP increased by 5.1 percentage points.

From 1990 to 2015, Mean Patch Area on private lands decreased by 26.4% for prairie and 23.8% for cropland, but increased by 79% for CRP (Table 2). Since 1990, Total Edge increased by 6.6% for prairie and 12.2% for cropland, while increasing in CRP by 62.8% (Table 2). Number of Patches in the ecoregion increased by 18.7% for prairie, 30.7% for cropland, and 30.7% for CRP since 1990 (Table 2). Clumpiness decreased by only 0.7% and 0.6% for prairie and cropland, respectively, since 1990. Clumpiness of CRP increased by 6.5% (Table 2). Contagion decreased by only about 3.4% from 1990–2015 (Table 2).

**Table 2:** Landcover metrics. FRAGSTATS landcover metrics [53] for landcover types on private lands in the lesser prairie-chicken Sand Sagebrush Prairie Ecoregion [36] in Kansas, Colorado, and Oklahoma, USA, from 1985–2015.

***a. Prairie***						
Year	Total Area ^1^	Mean Patch Area^2^	Percentage of Landscape^3^	Total Edge ^4^	Number of Patches^5^	Clumpiness^6^^,^^7^
1985	1137390.21	NA	36.46	NA	NA	NA
1990	1150138.35	35.89	36.87	45300.63	32045	0.95
1995	1140711.66	35.69	36.57	44545.20	31965	0.95
2000	1117750.41	34.91	35.83	43390.62	32022	0.95
2005	1100248.11	32.34	35.27	44068.02	34019	0.95
2010	1077971.94	29.34	34.56	45385.02	36744	0.95
2015	1004313.42	26.40	32.20	48291.99	38037	0.95

***b. Cropland***						
Year	Total Area ^1^	Mean Patch Area^2^	Percentage of Landscape^3^	Total Edge ^4^	Number of Patches^5^	Clumpiness^6^^,^^7^
1985	1938710.52	NA	62.15	NA	NA	NA
1990	1803324.51	68.56	57.81	46448.13	26301	0.95
1995	1776380.22	74.90	56.95	43720.23	23717	0.96
2000	1767507.93	77.37	56.66	41892.81	22844	0.96
2005	1713267.90	67.72	54.92	43025.94	25299	0.96
2010	1712343.87	56.75	54.89	46399.77	30171	0.95
2015	1796846.04	52.28	57.60	52092.48	34367	0.95

***c. CRP***						
Year	Total Area ^1^	Mean Patch Area^2^	Percentage of Landscape^3^	Total Edge ^4^	Number of Patches^5^	Clumpiness^6^
1990	118588.59	2.68	3.80	29999.40	44283	0.80
1995	154098.27	3.60	4.94	32320.20	42852	0.83
2000	185531.94	4.42	5.95	34597.17	41967	0.85
2005	260312.76	5.46	8.34	43571.58	47637	0.86
2010	284180.22	5.31	9.11	49183.47	53560	0.86
2015	278266.32	4.81	8.92	48853.47	57875	0.85

***d. All patch types (landscape level)***	
**Year **	**Contagion**^1^
1990	74.75
1995	74.06
2000	73.56
2005	72.32
2010	71.86
2015	72.24

1. Total area, in hectares, of all the patches combined for a given cover class in the landscape.

2. Average size, in ha, of patches within a given cover class in the landscape.

3. Proportion of the landscape within the study area consisting of a given cover class.

4. Total length of edge, in km, of all patches in a given cover class in the landscape.

5. Number of patches (groups of connected cells of the same cover class) for a given cover class on the landscape.

6. Degree of aggregation of cells in a given cover class in the landscape.

7. For consistency in comparisons following Conservation Reserve Program establishment in 1986, Mean Patch Area, Number of Patches, and Clumpiness were excluded from 1985 time step.

1. Contagion reflects both dispersion and interspersion of all patch types in the landscape [53] and is used here as a reflection of landscape-level fragmentation.

**Fig 6:**
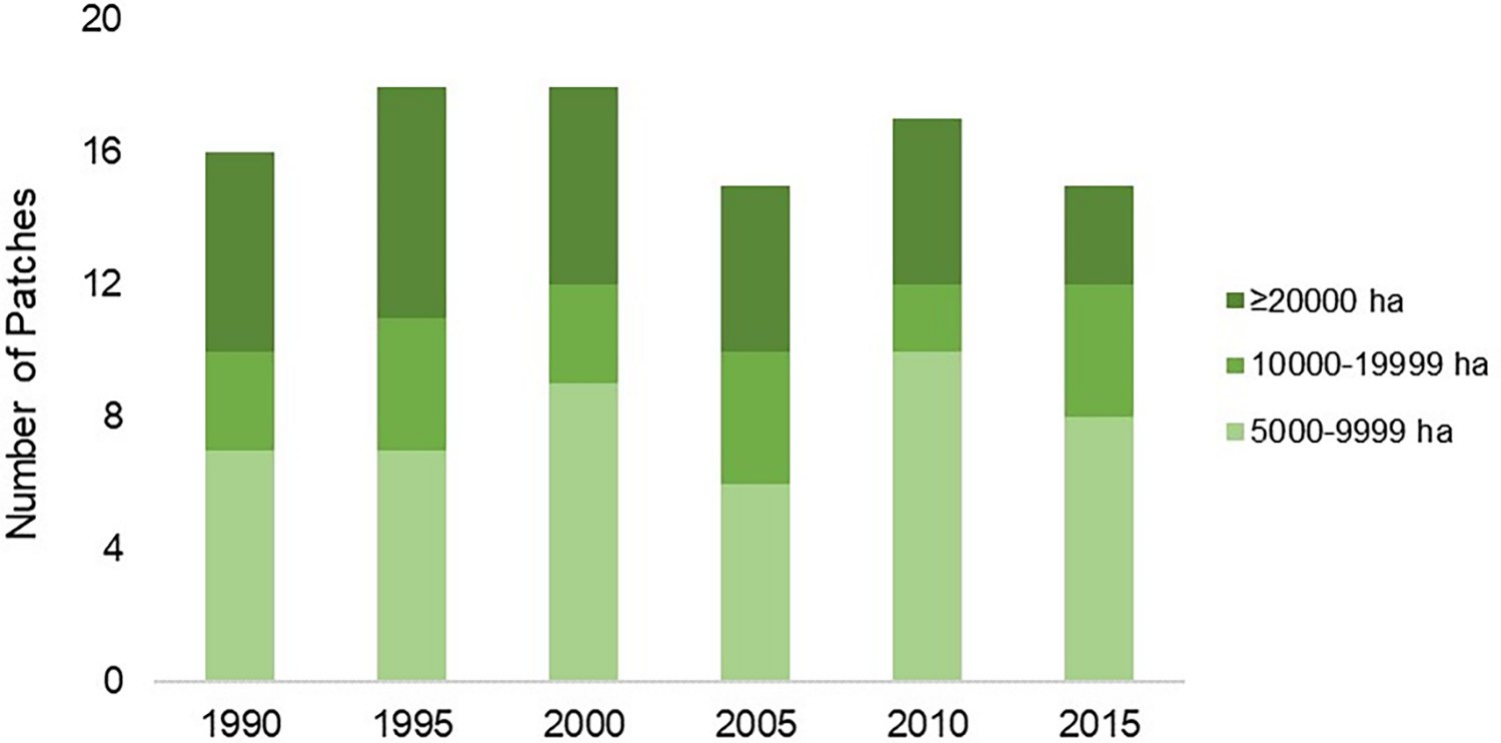
Large prairie patches. The number of large prairie patches (ranging from 5,000 to ≥20,000 ha) necessary to support lesser prairie-chicken populations in the Sand Sagebrush Prairie Ecoregion of southwestern Kansas, southeastern Colorado, and northwestern Oklahoma, USA, from 1990–2015.

The number of patches in all large patch size classes (from 5,000 ha through ≥20,000 ha) fluctuated from 1990–2015, but patches ≥20,000 ha decreased by 57% from 1990–2015 (Fig 6).

**Fig 7:**
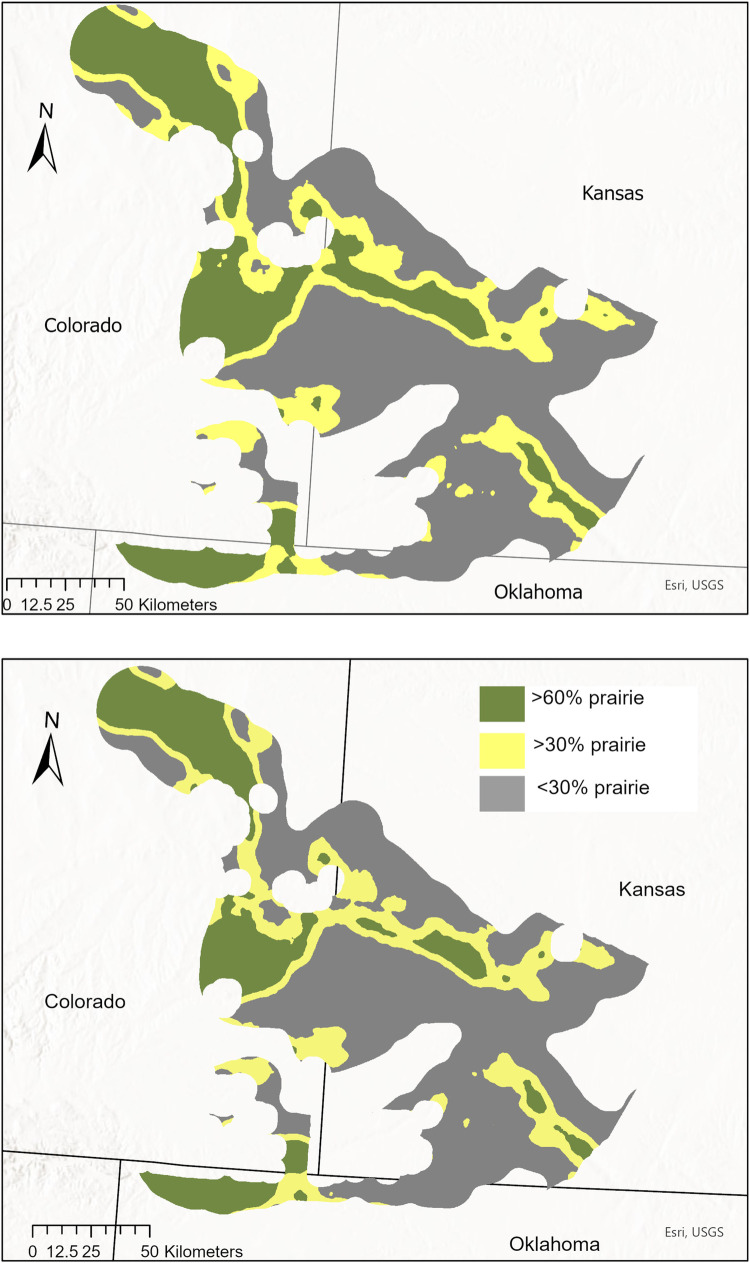
Percent prairie on private lands. Comparison of private lands areas in the Sand Sagebrush Prairie Ecoregion in Kansas, Colorado, and Oklahoma, USA, composed of <30%, >30% and >60% prairie within 5 km, respectively, during 1990 (top) and by 2015 (bottom). Areas with >60% prairie represent the recommended minimum amount of prairie for supporting lesser prairie-chicken populations [24].

In 1990, approximately 25% of private lands in the Sand Sagebrush Prairie Ecoregion consisted of “>60% prairie” at the 5-km scale, which decreased to approximately 20% by 2015 (Table 3, Fig 7), with most of the remaining area occurring in Colorado. Private lands with “>30% prairie” decreased by 5 percentage points from 1990–2015. Private lands with “>60% prairie” decreased by 4 percentage points from 1990–2015; a loss of approximately 92,544 ha.

**Table 3:** Percent prairie within 5 km. Percent of private lands in the Sand Sagebrush Prairie Ecoregion of Kansas, Colorado, and Oklahoma, USA, that consist of ≥30% and ≥60% prairie within 5-km windows from 1985–2015, based on moving window analysis.

	Percent of Private Lands		Total ha	
Year	>30% prairie	>60% prairie	>30% prairie	>60% prairie
1985	45.30	24.63	926732.07	503941.59
1990	45.77	24.87	936424.98	508844.88
1995	45.46	24.65	930068.55	504331.29
2000	44.76	24.09	915772.86	492829.02
2005	44.18	23.37	903779.19	478111.32
2010	43.49	22.82	889704.18	466801.29
2015	40.50	20.35	828574.47	416300.49

## Discussion

Our findings supported our hypotheses that availability of lesser prairie-chicken habitat in the Sand Sagebrush Prairie Ecoregion has changed over time at a broad scale. While the apparent decrease in cropland differed from our initial hypothesis, the decrease was concurrent with cropland conversion to CRP. The number of vertical anthropogenic structures did in fact increase, each potentially representing loss of available habitat due to avoidance [3,25,30,31]. Additionally, contiguous prairie-dominated areas have decreased in the ecoregion over time, as have the number of large prairie patches capable of supporting lesser prairie-chicken population persistence [13,24–26]. The combined effects of physical habitat loss and unavailability due to avoidance of structures may have had an additive effect on lesser prairie-chickens, potentially resulting in population declines to the degree observed since the mid-1980s.

Our finding that total grassland cover (combined prairie and CRP) increased marginally in the Sand Sagebrush Prairie Ecoregion is consistent with previous research showing that CRP offset grassland losses in the current lesser prairie-chicken range in Kansas [14]. It is unlikely that change in landcover totals was a primary contributor to population declines; however, increase in CRP may have contributed to shaping distribution of lesser prairie-chickens in the Sand Sagebrush Prairie Ecoregion [25,28].

While total prairie cover in the SSPE declined minimally since 1985, losses occurred in prairie areas with the greatest potential for supporting lesser prairie-chicken populations. The loss of ≥20,000 ha prairie patches and isolation of areas with >60% prairie occurred in the study area. The change in proportion of “>60%” prairie could indicate a threshold effect occurring in the Sand Sagebrush Prairie Ecoregion, with a baseline level of habitat incrementally lost in the time since population peak, eventually resulting in a pronounced and disproportionate population response [54,55].

Large, intact grassland-dominated landscapes are recommended for promoting stable lesser prairie-chicken populations [3,19,36]. While our landcover configuration metrics alone may suggest lack of fragmentation, the change in total prairie area together with findings of increasing edge, loss of the largest prairie patches, and decline in prairie-dominated areas on the landscape indicates that fragmentation of lesser prairie-chicken habitat has in fact occurred in the Sand Sagebrush Prairie Ecoregion. Smaller patches of prairie, or even isolated CRP fields not bounded by native prairie, are less likely to provide the heterogeneous cover needed for each phase of the lesser prairie-chicken’s life history including nesting, brooding, nonbreeding, and shelter from extreme weather [52,56].

The implications of change in available habitat are yet more concerning when considered with the effect of anthropogenic structures on functional loss of habitat. Our results show that parts of the ecoregion once free of major transmission lines and other features during the lesser prairie-chicken population peak are now populated by new vertical structures. Given anthropogenic structure avoidance by lesser prairie-chickens during nesting [48,49], in home range placement [31], and during lekking [50], increased structures may have essentially created zones of “non-habitat” or inaccessible habitat in the ecoregion, even in cover types otherwise conducive to lesser prairie-chickens such as native prairie [3,31,25]. Lesser prairie-chickens are documented crossing under transmission lines less frequently than would be expected at random, indicating vertical structures may contribute to population isolation by limiting dispersal [57]. Increasing structures may have disrupted connectivity among life-stage-specific habitat, and limited dispersal among prairie patches, ultimately affecting gene flow among local populations [11]. Continued development in areas free of vertical structures could further reduce available habitat throughout the Sand Sagebrush Prairie Ecoregion.

Broad-scale changes may be responsible for population declines of lesser prairie-chickens observed in recent decades, but it is unlikely these changes are the only contributing factor. Grassland vegetation composition and structure on public land managed for lesser prairie-chickens, particularly the U.S. Forest Service Cimarron and Comanche National Grasslands, should reflect characteristics indicative of quality lesser prairie-chicken habitat. However, recent data indicate that current vegetation on Cimarron and Comanche National Grasslands falls short of reproductive habitat recommendations [28]. It may be necessary to explore changes in lesser prairie-chicken habitat at finer scales to determine whether such changes may have an additive effect with broad-scale factors in driving lesser prairie-chicken population trends in the Sand Sagebrush Prairie Ecoregion.

The availability of CRP shows promise to increase reproductive habitat and population persistence for lesser prairie-chickens in the western portion of the species range [52]. In fact, establishment of native grass plantings in Kansas CRP may have promoted lesser prairie-chicken expansion and population increase north of the Arkansas River [58,59]. Extensive CRP use by lesser prairie-chickens has been documented in the Sand Sagebrush Prairie Ecoregion, particularly in eastern Colorado [19]. Lesser prairie-chickens in the ecoregion select for CRP over native prairie for nesting habitat [28]. However, given the uncertain future of CRP contracts as a long-term source of grassland cover, maintaining existing areas of connected prairie with low anthropogenic structure densities will remain essential for avoiding extirpation of lesser prairie-chickens in the Sand Sagebrush Prairie Ecoregion [21].

Our objectives in this broad-scale assessment were to determine the extent of landscape-level changes in lesser prairie-chicken habitat in the Sand Sagebrush Prairie Ecoregion since the contemporary population peak, and identify the potential for landscape-level changes negatively affecting the ecoregion’s capacity to support lesser prairie-chickens. The Sand Sagebrush Prairie Ecoregion has in fact changed since the mid-1980s, both in terms of widespread anthropogenic structures and in loss of contiguous native prairie on private lands. These pronounced changes, which occurred concurrently with observed population declines, likely reduced the capacity of the Sand Sagebrush Prairie Ecoregion to support persistence of lesser prairie-chicken populations. Our findings support recent estimates suggesting that only 9% of the Sand Sagebrush Prairie Ecoregion remains as potential habitat for lesser prairie-chickens when accounting for anthropogenic structures and cover composition [25]. Under current conditions, lesser prairie-chicken populations in the Sand Sagebrush Prairie Ecoregion are unlikely to return to densities observed in the mid-1980s.

Focusing management efforts on preventing further habitat losses in the remaining potential habitat, as well as promoting connectivity among extant populations, may minimize continued population declines. It may also be necessary to explore finer-scale factors in lesser prairie-chicken habitat, including changes in vegetation composition and structure over time, to supplement our broad-scale findings.

## Supporting information

S1 File Colorado and Oklahoma towers in the Sand Sagebrush Prairie Ecoregion. (CSV)

S2 File Kansas cell towers in the Sand Sagebrush Prairie Ecoregion. (CSV)

S3 File Kansas oil and gas wells in the Sand Sagebrush Prairie Ecoregion. (CSV)

S4 File Oklahoma oil and gas wells in the Sand Sagebrush Prairie Ecoregion. (CSV)

S5 File Colorado oil and gas wells in the Sand Sagebrush Prairie Ecoregion. (CSV)

S6 File Transmission lines in the Sand Sagebrush Prairie Ecoregion. (CSV)

S7 File Wind turbines in the Sand Sagebrush Prairie Ecoregion. (CSV)

S8 File Creating a public lands shapefile. (PDF)

S9 File Calculating CRP from LCMAP rasters. (PDF)

S10 File Estimating percent prairie. (PDF)
