# Supplementary material for: Broad-scale changes in lesser prairie-chicken habitat
Source: PLoS One. 2024 May 31;19(5):e0304452. doi: 10.1371/journal.pone.0304452 (PMC11142685; doi:10.1371/journal.pone.0304452)
Supplement: S8 File — (PDF) [file pone.0304452.s008.pdf]

We obtained CO public lands shapefiles from publicly-available GIS data of Colorado Parks and Wildlife property boundaries (https://www.arcgis.com/home/item.html?id=b1b27dc4bde744e490e0d1a9f9512032)

and Bureau of Land Management lands (https://gbp-blm-egis.hub.arcgis.com/pages/colorado). We combined public lands for Colorado and Kansas, including Cimarron and Comanche National Grasslands boundaries, into one shapefile and used the resulting public lands shapefile to create a "private lands" USGS Land Change Monitoring, Assessment, and Projection (LCMAP) raster.

Any use of trade, firm, or product names is for descriptive purposes only and does not imply endorsement by the U.S. Government.
