# Supplementary material for: Broad-scale changes in lesser prairie-chicken habitat
Source: PLoS One. 2024 May 31;19(5):e0304452. doi: 10.1371/journal.pone.0304452 (PMC11142685; doi:10.1371/journal.pone.0304452)
Supplement: S9 File — (PDF) [file pone.0304452.s009.pdf]

In Raster Calculator for every pair of time steps, we used the Map Algebra expression: "([layer for year a]*1000) + [layer for year b])" to yield a history of change for land cover types. We created a landcover change raster for each time step ([bounded by 2 LCMAP rasters, 5 years apart]) in Raster Calculator in ArcGIS Pro, which yielded rasters with categories for "2002" (started as crops, or category "2", and remained as cropland through end of time step), "2003" (crop that changed to grass/shrub, or "3", cover type), etc. We did this to estimate change between consecutive time steps. Classifying the land cover history in this manner would identify which cover types remained the same, and which cover types changed during the 5 -year interval – with the objective of identifying specifically "2003" coded cover categories; that is, crop (2) that changed to apparent grass/shrub (3). For this reason, for each of the 2 landcover change rasters we created another Raster Calculator [binary raster] showing only "2003" (crop to new grass/forb) as 1, and everything that was not "2003" designated as 0. This would identify crop that was changed into grass at a given time step; that is, new CRP.

Next in Raster Calculator, we built the expression: " Con((("estimatedCRP_8590_2.tif" ==1) & ("Clip_LCMAP_CU_1995_V01_LCPRI_MinusPublic" ==3)) | ("binrasCRP9095" ==1),1,0)" to yield all grid cells identified as "2003" (or crop to grass; that is, CRP) from the previous time step that would also be identified in LCMAP as grass/shrub ("3") through 1995 – this finds all CRP but does not include expired fields turned back to cropland; in other words, a summary of non-expired CRP as of 1995. This expression also asks to include anything with code "2003" (crop that changed to grass; theoretically, CRP) in the next consecutive time step (here, 1990-95). This results in a product that includes all the previously identified, non-expired CRP, plus new enrollment. To incorporate the newly estimated CRP pixels into the LCMAP raster categories, we built a statement to yield a raster made up of "2003" (CRP) pixels, with the

LCMAP pixels in place of wherever there is “non CRP”: Con("estimatedCRP_8590_2.tif" == 1 ,9,"Clip_LCMAP_CU_1990_V01_LCPRI_MinusPublic").

We summed the total number of pixels of each cover type of interest (i.e., cropland, prairie, CRP) for each time step and converted to hectares.

Any use of trade, firm, or product names is for descriptive purposes only and does not imply endorsement by the U.S. Government.
