# Supplementary material for: Broad-scale changes in lesser prairie-chicken habitat
Source: PLoS One. 2024 May 31;19(5):e0304452. doi: 10.1371/journal.pone.0304452 (PMC11142685; doi:10.1371/journal.pone.0304452)
Supplement: S10 File — (PDF) [file pone.0304452.s010.pdf]

We created a set of binary rasters (1=grass/shrub, 0=everything else, noData=NoData) based on using my “LCMAP [year] plus CRP, filtered” named series of layers. I ran each through Focal Statistics in ArcGIS Pro with the following parameters: circle, 5000, Map; and unchecked the box to “Ignore NoData in calculations”. We unchecked “Ignore NoData”, otherwise the analysis would extrapolate over the “cut out” public lands.

We plugged the Focal Statistics rasters into Raster Calculator specifying “[raster name for given year]>.3”, and separately, “[raster for that given year]>.6”, and recorded the count value associated with the “1” category for each raster. We identified only >30% and > 60%, but used Mosaic to New Raster to create a single raster of “0,1,2” categories for each time step.

Any use of trade, firm, or product names is for descriptive purposes only and does not imply endorsement by the U.S. Government.
